# Diagnostic Errors in Initial Misdiagnosis of Foreign Body Aspiration in Children: A Retrospective Observational Study in a Tertiary Care Hospital in China

**DOI:** 10.3389/fped.2021.694211

**Published:** 2021-10-15

**Authors:** Yingchao Zhu, Qijun Fan, Lijun Cheng, Bobei Chen

**Affiliations:** ^1^Department of Otolaryngology, The Second Affiliated Hospital and Yuying Children's Hospital of Wenzhou Medical University, Wenzhou, China; ^2^The Second School of Medicine, Wenzhou Medical University, Wenzhou, China

**Keywords:** pediatric, foreign body, misdiagnosis, aspiration, airway, bronchoscopy, medical management

## Abstract

**Background:** Foreign body aspiration (FBA) in children is a common emergency that can easily be missed, leading to delays in treatment. Few large cohort studies have focused on errors in diagnostic assessment. The main purpose of this study was to analyze factors contributing to the initial misdiagnosis of FBA in children.

**Methods:** We retrospectively reviewed the charts of 226 children diagnosed with FBA at the Second Affiliated Hospital and Yuying Children's Hospital of Wenzhou Medical University from January 2018 to November 2020. Cases were divided into two groups according to whether or not patients were initially misdiagnosed. The clinical characteristics of the two groups were then compared. The Diagnosis Error Evaluation and Research (DEER) taxonomy tool was applied to cases with initial misdiagnosis.

**Results:** Of the 226 included children with a final diagnosis of FBA, 153 (67.7%) were boys. Ninety percent of patients were under 3 years old. More than half (61.9%) of the children were referred from primary institutions, and 38.1% visited tertiary hospitals directly. A total of 80 (35.4%) patients were initially misdiagnosed. More than half of misdiagnosed children received an alternative diagnosis of bronchiolitis (51.3%), the most common alternative diagnosis. Test failures (i.e., errors in test ordering, test performance, and clinician processing) were primarily responsible for the majority of initial diagnostic errors (76.3%), followed by failure or delay in eliciting critical case history information (20.0%). Characteristics significantly associated with initial misdiagnosis were: presentation over 24 h (OR 9.2, 95% CI 4.8–17.5), being referred from primary institutions (OR 8.8, 4.1–19.0), no witnessed aspiration crisis (OR 7.8, 3.0–20.3), (4) atypical signs or symptoms (OR 3.2, 1.8–5.7), foreign body not visible on CT (OR 36.2, 2.1–636.8), foreign body located in secondary bronchi (OR 4.8, 1.3–17.2), organic foreign body (OR 6.2, 1.4–27.2), and history of recurrent respiratory infections (OR 2.7, 1.4–5.3). Children with misdiagnosis tended to have a longer time from symptom onset to the definitive diagnosis of FBA (*P* < 0.001).

**Conclusions:** More than one-third of children with FBA were missed at first presentation. Errors in diagnostic testing and history taking were the main reasons leading to initial misdiagnosis.

## Introduction

In children, foreign body aspiration (FBA) is a potentially common emergency that may threaten the patient's life (1, 2). It occurs mostly in males younger than 3 years of age (3). The initial clinician experience in recognizing FBA is critical for timely diagnosis (4). Witnessed aspiration crisis and the triad of cough, wheeze, and diminished breath sounds are specific in establishing clinical suspicion of FBA (5, 6). However, some patients present with atypical symptoms that may be ignored (6). Therefore, in clinical practice, radiologic examinations and bronchoscopy are important to assist clinicians with diagnosis. Computed tomography (CT) is regarded as the optimal diagnostic tool as it is superior to the chest X-ray and less invasive than bronchoscopy (7). FBA may lead to complications or fatal damage if left untreated. Hence, it is essential to make the correct diagnosis early and extract the foreign body (FB) with the least delay.

The Diagnosis Error Evaluation and Research (DEER) classification system is a tool to categorize diagnostic errors by the location in the diagnostic process where a problem occurred (8).

Previous studies have researched clinical characteristics, diagnostic and therapeutic methods, and the hazard of delayed treatment, but few large-scale studies focus on errors in diagnostic assessment (4, 9). Therefore, we tried to identify the factors contributing to the misdiagnosis of FBA to minimize diagnostic errors and increase the probability of a favorable prognosis.

## Materials and Methods

This study was approved by the Second Affiliated Hospital of Wenzhou Medical University review board. We retrospectively reviewed all cases of FBA from January 2018 to November 2020. Electronic and paper medical records were examined to identify patients with the final diagnosis of FBA. We included all children referred to us with a prior diagnosis of FBA or who received a diagnosis of FBA in our institution. Experienced Otorhinolaryngologists determined the diagnosis of FBA based on surgical findings or objects coughed up. Cases where diagnosis was undetermined were excluded. Demographic data, including age, sex, and BMI, were collected. We also retrieved patient history, initial symptoms, examination findings, diagnostic testing, and treatments. Detailed referral data, including the time to the final diagnosis and specialty of clinicians seen previously, were also recorded. Cases were divided into 2 groups according to whether or not patients were misdiagnosed. The clinical characteristics of the two groups were then compared. We used the DEER taxonomy to classify each case with a missed diagnosis according to the location and type of error in the diagnostic process. When there were multiple errors in the process, the major cause of misdiagnosis was categorized as a primary DEER category, and other breakdowns were assigned as secondary or tertiary (10). As the tool is subjective, the DEER classification was independently assigned by 3 experienced experts (Q.F., L.C., and B.C.), with differences resolved by consensus.

Statistical analyses were performed using SPSS 26.0 (IBM Corporation, Armonk, New York). The median and interquartile range (IQR) were reported for continuous measures, as they were not normally distributed. Comparisons between the group with and without misdiagnosis were conducted using the Mann-Whitney U test. Percentages are reported for categorical measures, and the comparisons were conducted using either the χ^2^ test or Fisher's exact test. When one or more cells in a data table have a value of zero, Haldane s correction was employed. Odds Ratios (OR) with 95% confidence intervals (CI) was assessed in two groups. All *P*-values were 2-tailed, and statistical significance was defined as *P* < 0.05.

## Results

**Figure 1:**
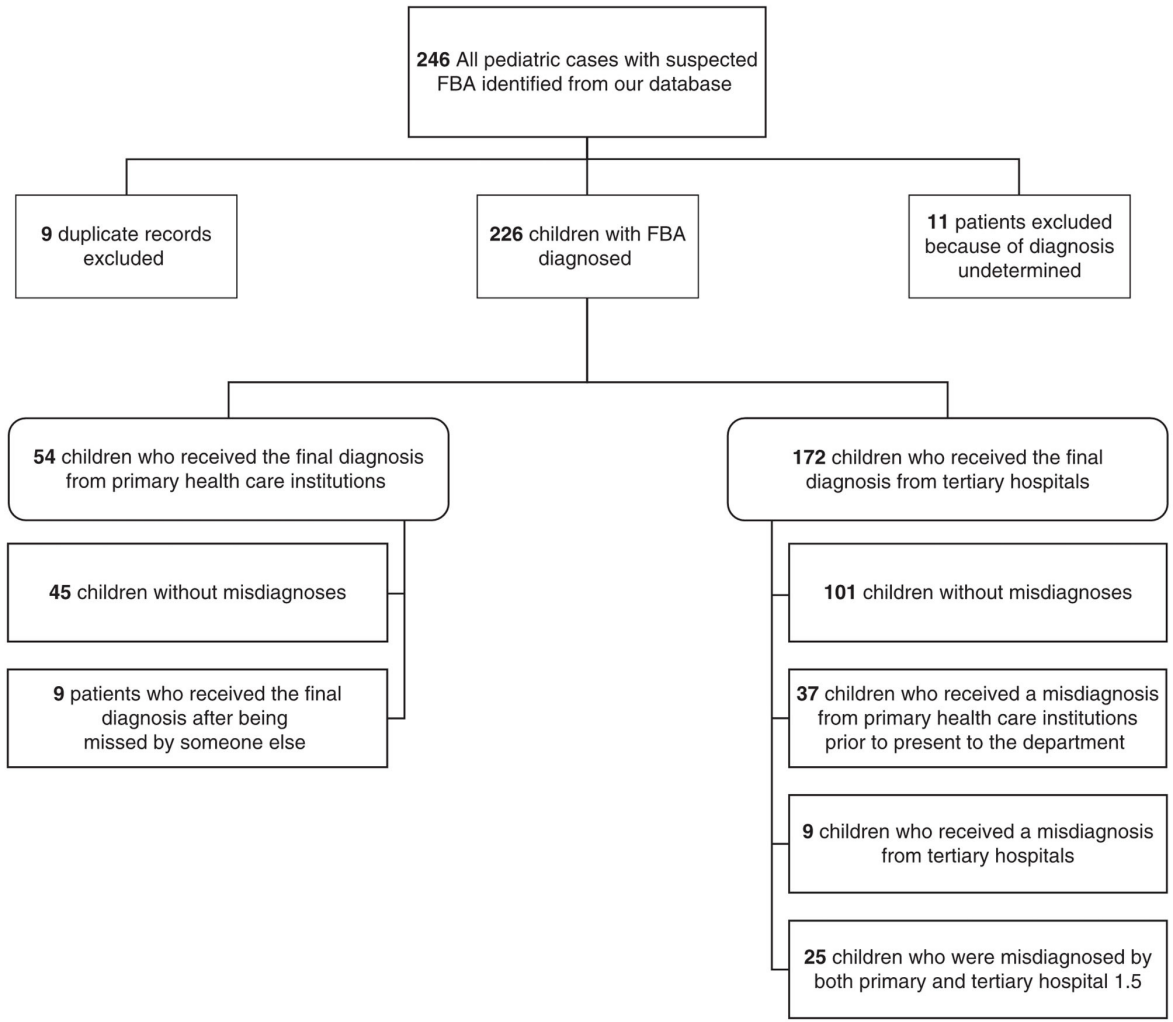
Classification of patients according to the diagnosis pattern.

A total of 237 pediatric patients with suspected FBA were identified from our database after excluding 9 duplicate records. Because our primary concern was to analyze the process of diagnosis, 11 patients with undetermined diagnoses were excluded. The remaining 226 cases were diagnosed by primary health care institutions (*n* = 54) or tertiary hospitals (*n* = 172). The detailed classification of patients according to the diagnosis pattern is shown in Figure 1.

Of the total sample, 153 children were boys (67.7%), and 73 (32.3%) were girls. The median age was 18.8 months (range: 6–130 months). Ninety percent of patients (204 cases, 90.3%) were 3 years or younger. Most (61.9%) children were referred from primary health care institutions, and 38.1% visited tertiary hospitals directly. The time between discomfort and presentation ranged from 1 h to 210 days with a median of 1.0 days. Twenty-six (11.5%) patients were without witnessed aspiration history (Table 1).

**Table 1:** Characteristics of study patients.

	***n*** **(%)**
**Sex**	
Male	153 (67.7)
Female	73 (32.3)
**Age at admission**	
< 1 year	19 (8.4)
1–3 years	185 (81.9)
>3 years	22 (9.7)
**Duration of symptoms before presentation**	
<24 h	121 (53.5)
>24 h	105 (46.5)
**Site of initial presentation**	
Tertiary institutions	86 (38.1)
Primary institutions	140 (61.9)
**History of witnessed aspirated FB (+)**	200 (88.5)
**Typical signs or symptoms (+)**	151 (66.8)
**CT findings**	
Foreign body (+)^※^	204 (96.2)
**Location of foreign body**	
Larynx and trachea	22 (9.7)
Left primary bronchus	78 (34.5)
Right primary bronchus	92 (40.7)
Bilateral primary bronchus	1 (0.4)
Left secondary bronchi	14 (6.2)
Right secondary bronchi	19 (8.4)
**Type of foreign body**	
Organic	204 (90.3)
Inorganic	22 (9.7)
**Past medical history**	
Asthma	5 (2.2)
Recurrent respiratory infection	44 (19.5)
Language developmental disorder	3 (1.3)
**Outcome**	
Complete recovery after first bronchoscopy	203 (89.8)
Complete recovery after repeat bronchoscopy	10 (4.4)
Referred to ICU with critical condition after bronchoscopy	8 (3.5)
Died before an operation	2 (0.9)
Coughed up	3 (1.3)

¸ *Number of available CT (n = 212)*.

A total of 151 (66.8%) children had at least one clinical sign and symptom with diagnostic value (such as choking, noisy breathing/stridor/dysphonia, cyanosis, new onset wheezing/recurrent/persistent wheeze, or unilateral reduced air entry), and 75 cases (33.2%) only had atypical signs and symptoms overlapping with other common pediatric conditions (such as cough, vomiting, fever, tachypnea, and crackles) (11). The CT scan was a conventional pre-operative examination and highly effective, with a 96.2% (204/212) detection rate. The majority of the foreign bodies were organic (204, 90.3%), with peanuts (74, 32.7%) being the most widespread. Inorganic objects (such as plastic and metal materials) accounted for 9.7%. The right primary bronchus (92, 40.7%) was the most frequently identified location, followed by the left primary bronchus (78, 34.5%) and the main airway (including the larynx and trachea) (22, 9.7%). One child's FB was in both bronchial trees. Forty-four (19.5%) children presented with a history of recurrent respiratory infection. Most (210, 92.9%) FBs were successfully extracted via rigid bronchoscopy or flexible bronchoscopy at the first session, and 11 cases underwent repeat bronchoscopy. Three patients coughed up FBs, and 2 children died before they could be operated on. Eight children were referred to ICU in critical condition (Table 1).

A total of 80 (80/226, 35.4%) patients experienced at least one error in the diagnostic assessment. Before the final diagnosis, 50 of the misdiagnosed children had been evaluated by GPs, 22 by pediatric respirologists, 5 by Emergency Medicine (EM) clinicians, and 3 by ENT specialists.

More than half the misdiagnosed children received an alternative diagnosis of bronchiolitis (41, 51.3%), the most common alternative diagnosis. Another common misdiagnosis was pneumonia (32, 40.0%). Less frequent misdiagnoses included asthma (3, 3.8%), bronchiectasis (2, 2.5%) and acute laryngitis (2, 2.5%).

The DEER taxonomy tool categorized factors contributing to diagnostic error. Testing and history errors were the most common factors. Test failures (errors in radiology ordering, performance, and clinician processing) were primarily responsible for the major diagnostic errors in 61 of the 80 cases (76.3%), mainly consisting of 34 cases with a failure or delay in ordering needed tests (CT or bronchoscopy; Chest X-ray is defined as a test needed for patients with radiopaque foreign body aspiration history) and 18 cases with an error in clinician interpretation of test (such as a failure in recognizing FB or misinterpreting FB as a mucus plug). The failure or delay in eliciting critical aspects of the history data (especially aspiration history denied by parents or delay in providing witnessed history) was the only factor contributing to history error (16, 20.0%). Three patients (3.8%) received a misdiagnosis because of clinician assessment failures, associated with placing too much weight on a competing diagnosis (Table 2).

**Table 2:** Diagnosis error evaluation and research taxonomy.

**Point in the diagnostic process (anatomic localization)**	**What went wrong (lesion)**	**No. (%)**	
		**Patients (*n* = 80) with diagnostic errors (*n* = 221) in each category**	**Patients (*n* = 80) with the primary diagnostic error (*n* = 80) in each category**
1. Access/presentation			
A	Failure/delay in presentation		
B	Failure/denied care access		
2. History		35 (43.8)	16 (20.0)
A	Failure/delay in eliciting critical piece of history data	18	16
B	Inaccurate/misinterpretation	2	
C	Failure in weighing	15	
D	Failure/delay to follow-up		
3. Physical examination		26 (32.5)	
A	Failure/delay in eliciting critical examination finding		
B	Inaccurate/misinterpreted	11	
C	Failure in weighing	15	
D	Failure/delay to follow-up		
4. Tests		73 91.3	61 76.3
Ordering			
A	Failure/delay in ordering needed test(s)	41	34
B	Failure/delay in performing ordered test(s)		
C	Error in test sequencing		
D	Ordering of the wrong test(s)		
E	Test ordered the wrong way	13	9
Performance			
F	Sample mix-up/mislabeled (e.g., wrong patient/test)		
G	Technical error/poor processing of specimen/test		
H	Erroneous laboratory/radiology reading of test		
I	Failed/delayed reporting of the result to the clinician		
Clinician processing			
J	Failed/delayed follow-up (abnormal) test result		
K	Error in clinician interpretation of test	19	18
5. Assessment		71 88.8	3 3.8
Hypothesis generation			
A	Failure/delay in considering the correct diagnosis	13	
Suboptimal weighing/prioritizing			
B	Too little consideration/weight given to the diagnosis	27	
C	Too much weight on competing/coexisting diagnosis	31	3
Recognizing urgency/complications			
D	Failure/delay to recognize/weigh urgency		
E	Failure/delay to recognize/weigh complication(s)		
6. Referral/consultation		16 20.0	
A	Failure in ordering referral	16	
B	Inappropriate/unneeded referral		
C	Error in diagnostic consultation performance		
D	Failed/delayed communication/follow-up of consultation		
7. Follow-up			
A	Failure to refer patient to close/safe setting/monitoring		
B	Failure/delay in timely follow-up/rechecking of patient		

When all the errors—including secondary and tertiary—were considered, 221 diagnostic breakdowns were found. Most errors were due to testing failures (73 of 221, 33.0%) and failures in clinician assessment (71 of 221, 32.1%). In conclusion, 35 of the 80 misdiagnosed patients (43.8%) involved history misinterpretation; 26 patients (32.5%) experienced errors in physical examination; and 73 cases (91.3%) were subject to testing errors, a similar proportion to the cases involved in clinician assessment failure (71, 88.8%). Sixteen patients (20.0%) experienced inappropriate referrals. When we performed a subgroup analysis of the errors, we found that failures in ordering needed tests occurred in more than half (41, 51.3%) of the cases, followed by the overvaluation of competing diagnoses (31, 38.8%) and underestimation of the possibility of FBA (27, 33.8%) (Table 2).

When pediatric cases with or without misdiagnosis were compared, children without diagnostic breakdown tended to have less time from symptom onset to presentation (OR 9.2, 95% CI 4.8–17.5, *P* < 0.001). Misdiagnosis was more common in patients without witnessed aspiration crisis (OR 7.8, 95% CI 3.0–20.3, *P* < 0.001). In addition, being referred from primary institutions (OR 8.8, 95% CI 4.1–19.0, *P* < 0.001), a history of recurrent respiratory infections (OR 2.7, 95% CI 1.4–5.3, *P* = 0.005) and atypical signs or symptoms (OR 3.2, 95% CI 1.8–5.7, *P* < 0.001) were each associated with misdiagnosis. FBs of misdiagnosis cases were more likely organic (OR 6.2, 95% CI 1.4–27.2, *P* = 0.008), invisible on CT (OR 36.2, 95% CI 2.1–636.8, *P* < 0.001) and located in secondary bronchi (OR 4.8, 95% CI 1.3–17.2, *P* = 0.022). The time elapsed between symptoms and final diagnosis was significantly longer among patients with misdiagnoses (Table 3).

**Table 3:** Demographic and clinical characteristics of patients with correct and missed diagnoses of FBA.

	**Missed (*n* = 80)**	**Not missed (*n* = 146)**	**OR (95% CI)**	***P*** **-value**
Duration of symptoms before presentation, *n* (%)				**<0.001**
<24 h	17	104	Reference	
>24 h	63	42	9.2 (4.8–17.5)	
Site of initial presentation, *n* (%)				**<0.001**
Tertiary institutions	9 (11.3)	77 (52.7)	Reference	
Primary institutions	71 (88.8)	69 (47.3)	8.8 (4.1–19.0)	
History, *n* (%)				**<0.001**
Witnessed aspiration crisis	60 (75.0)	140 (95.9)	Reference	
No witnessed aspiration crisis	20 (25.0)	6 (4.1)	7.8 (3.0–20.3)	
Signs or symptoms, *n* (%)				**<0.001**
Typical	40 (50.0)	111 (76.0)	Reference	
Atypical	40 (50.0)	35 (24.0)	3.2 (1.8–5.7)	
CT findings^※^, *n* (%)				**<0.001**
Foreign body (+)	65 (89.0)	139 (100.0)	Reference	
Foreign body (–)	8 (11.0)	0 (0.0)	36.2 (2.1–636.8)	
Location of foreign body, *n* (%)				
Side				0.258
Middle	4 (5.0)	18 (12.3)	Reference	
Left	35 (43.8)	57 (39.0)	2.8 (0.9–8.8)	0.087
Right	41 (51.3)	70 (47.9)	2.6 (0.8–8.3)	0.137
Bilateral	0 (0.0)	1 (0.7)	1.4 (0.0–39.5)	1.000
Location				**0.035**
Larynx and trachea	4 (5.0)	18 (12.3)	Reference	
Primary bronchus	59 (73.8)	112 (76.7)	2.4 (0.8–7.3)	0.151
Secondary bronchi	17 (21.3)	16 (11.0)	4.8 (1.3–17.2)	**0.022**
Type of foreign body, *n* (%)				**0.008**
Inorganic	2 (2.5)	20 (13.7)	Reference	
Organic	78 (97.5)	126 (86.3)	6.2 (1.4–27.2)	
Past medical history, *n* (%)				
Recurrent respiratory infection	24 (30.0)	20 (13.7)	2.7 (1.4–5.3)	**0.005**
The time elapsed between symptoms and final diagnoses, *n* (%)				
Over 24 h	64 (80.0)	43 (29.5)	–	**<0.001**
Over 72 h	55 (68.8)	24 (16.4)	–	**<0.001**
Over 1 week	39 (48.8)	16 (11.0)	–	**<0.001**

*Part of results without statistical significance were omitted.*

*Values in bold indicate P < 0.05 on univariate analysis.*

※ *Number of available CT (n = 73, n = 139)*.

## Discussion

FBA is a serious and potentially fatal problem (12). Most FBs are safe when they lodge firmly in the bronchial tree, but consequences can be fatal when FBs move to the larynx or trachea and obstruct the main airway completely. Moreover, FBs lodged in the airway over an extended period can lead to other lung problems (4, 13, 14).

The National Academy of Medicine (NAM) defined diagnostic error as a “failure to establish an accurate and timely explanation of the patient's health problem(s) or communicate that explanation to the patient” (10, 15). The Committee emphasizes that “improving diagnosis is not only possible, but it also represents a moral, professional, and public health imperative” (10, 15). Thus, it is essential to analyze factors that lead to errors and to optimize diagnostic processes.

The study indicated that more than one-third (35.4%) of children with FBA received misdiagnoses. We applied the DEER taxonomy tool to evaluate errors further. This tool has recently been used in several high quality studies of misdiagnosis (8, 10, 16–18). Errors in diagnostic testing (65.0%) and eliciting critical elements of history (20.0%) were the main reasons leading to initially missed diagnoses. Assessment of secondary and tertiary errors indicate most cases were subject to testing errors (91.3%), similar to the cases involved in clinician assessment failure (88.8%).

The proportion of misdiagnoses made by tertiary institutions was significantly lower than those misdiagnosed by primary institutions in this study. A similar finding was reported by Hang, who suggested that inexperience of the primary care clinician and/or lack of adequate equipment contributed to significant delays in FB management (4). Generally speaking, clinicians who serve tertiary institutions are better trained than others and more likely to possess specialized knowledge (4). GPs need appropriate specialized training to improve their diagnostic acumen, and all initial clinicians should consciously acknowledge the risk of thinking in a silo and actively seek explanations outside their specialty (19).

Clinicians focus excessively on more commonly seen and previously diagnosed diseases (19). As this research demonstrated, the majority of children received a misdiagnosis of bronchiolitis or pneumonia. Clinicians with confirmation bias tended to interpret atypical symptoms and abnormal lung auscultation as simplex respiratory inflammation. As for cases with a history of recurrent respiratory infections, clinicians readily overweighted previous diagnoses and failed to consider FBA. This suggests that clinicians should rely on evidence-based and objective individual data, not only disease prevalence rates or an overconfident view (19).

Clinical suspicion of FBA mainly depended on the witnessed aspiration history, clinical symptoms and signs. However, unfortunately, a reliable history is not always available (20–23). In our review, 88.5% of patients' families provided a history of FBA, and the group without misdiagnosis had a significantly higher rate of positive aspiration history than the misdiagnosed group. Goyal et al. demonstrated that patients presenting within a week of onset often complain of wet cough, wheeze, and tachypnea, whereas those who present after a week have dry cough and fever as their primary complaint (23). Some patients are asymptomatic and present no alterations on physical examination (24). As the FB passes through the vocal cords into the lower airway, patients may be asymptomatic or present with non-specific symptoms and signs. During this time, the diagnosis may be more difficult to confirm. Medical attention may only be sought when patients have complications that mimic intermittent tracheobronchitis, recurrent pneumonia, or asthma (20). It is important to realize that the diagnostic criteria for FBA do not account for atypical disease manifestations, and the diagnosis of FBA is an iterative and interactive process that should not be prematurely concluded (1, 19).

Radiologic examination and bronchoscopy are pivotal parts of the initial evaluation. However, many new clinicians find it challenging to select and interpret assessments in an unbiased, situation-based way, fine-tuning for multiple factors. The chest X-ray was performed as the first-line radiographic investigation. A well-performed X-ray (inspiration and expiration) may yield good results for organic or small FBA during inspiration and expiration. However, paired inspiratory and expiratory films were not routinely feasible due to lack of cooperation (25). Compared with organic objects, metal materials were clearly revealed, but small organic food items that cannot be seen directly on X-ray are most common (3, 5, 20). Data from several studies suggest that the percentage of patients with FBA presenting no alterations on X-ray ranges from 10 to 46% (5). Thus, placing too much importance on simple X-ray as an examination for excluding FBA can lead to misdiagnosis (24). Based on this, we used CT as a pre-operative examination that could show the FB's anatomical location, shape, and size, and is helpful for operative planning (23, 26, 27). However, considering radiation exposure and cost, clinicians tend to avoid ordering CT for patients without witnessed aspiration history or typical clinical characteristics. Although bronchoscopy (including rigid and flexible bronchoscopy) is deemed the gold standard for diagnosis and the definitive therapeutic intervention for FBA, it remains an invasive procedure under anesthesia and carries a risk of complications. In practice, clinicians try to avoid unnecessary bronchoscopy in children (7). Inexperienced clinicians may struggle to weigh the benefits of bronchoscopy against the risks associated with it. Scoring systems can help support clinicians with their decision-making (11, 28, 29).

This study demonstrated that misdiagnosis of FBA may be associated with delayed diagnosis, a finding also reported by previous studies (20, 30). As we focused primarily on diagnostic assessment errors, limited attention was given to prognosis and complications. Previous studies of FBA have indicated that delayed diagnosis and treatment can increase the risk of complications (5, 30, 31).

There are limitations to this study. Our department is an authoritative pediatric tertiary center in south-eastern China accepting patients from neighboring areas. However, as a single-center study, it is unknown to what extent findings can be generalized to other geographic areas. We reviewed a relatively large number of cases, but when divided into subcategories, the numbers were smaller and less could be determined about the subcategories. Additionally, this study was limited by the accuracy of the information in the health records available. Most patients had electronic health records, although a few had paper medical records derived from electronic health records. The DEER taxonomy tool is subjective. When there was more than one error in the process of diagnosis, discussion took place about categorization of the primary DEER category (8). In each case, our experts evaluated independently and tried to identify the main reason for the misdiagnosis. The DEER taxonomy has been used before in other clinical scenarios, but this was the first study applying the DEER to FBA. Further work is required to discuss the adequacy of the DEER when used for different clinical research areas.

The fundamental purpose of this study was to identify pitfalls or biases that may contribute to a missed diagnosis of FBA. Test failures were mainly responsible for major diagnostic errors. Appropriate ordering and interpretation of tests, eliciting all critical case history information, and timely consideration given to the diagnosis of FBA will improve the diagnostic process and ensure delayed treatment is avoided.

## Data Availability Statement

The raw data supporting the conclusions of this article will be made available by the authors, without undue reservation.

## Ethics Statement

The studies involving human participants were reviewed and approved by The Second Affiliated Hospital of Wenzhou Medical University review board. Written informed consent from the participants' legal guardian/next of kin was not required to participate in this study in accordance with the national legislation and the institutional requirements.

## Author Contributions

YZ: conceptualization (lead), data curation (equal), investigation (equal), methodology (lead), writing—original draft preparation (lead), and writing—review and editing (equal). QF: formal analysis (lead), writing—original draft preparation (supporting), and writing—review and editing (equal). LC: data curation (equal), investigation (equal), writing—original draft preparation (supporting), and writing—review and editing (equal). BC: conceptualization (supporting), project administration (lead), resources (lead), supervision (lead), and writing—review and editing (equal). All authors contributed to the article and approved the submitted version.

## Funding

This research is supported by Science and Technology Plan Project of Wenzhou (Grant Nos: Y20180166, Y2020028, and Y20210014) and Science and Technology Plan Project of Taizhou (Grant No: 20ywb83). The funders had no role in the study design, data collection and analysis, decision to publish, or preparation of the manuscript.

## Conflict of Interest

The authors declare that the research was conducted in the absence of any commercial or financial relationships that could be construed as a potential conflict of interest.

## Publisher's Note

All claims expressed in this article are solely those of the authors and do not necessarily represent those of their affiliated organizations, or those of the publisher, the editors and the reviewers. Any product that may be evaluated in this article, or claim that may be made by its manufacturer, is not guaranteed or endorsed by the publisher.

## Abbreviations

FBA: Foreign Body Aspiration
FB: Foreign Body
DEER: Diagnosis Error Evaluation and Research
CT: Computed Tomography.
